# High voltage hobby; electrical burn following fractal wood burning: A case report

**DOI:** 10.1016/j.jpra.2025.02.019

**Published:** 2025-02-26

**Authors:** Hollie Moran, Eugene Koh, Elizabeth Concannon, Marcus Wagstaff

**Affiliations:** Burns Unit, Royal Adelaide Hospital, Adelaide, SA, Australia

**Keywords:** Burns, Electrical, Electricity, Fractals, Pyrography

## Abstract

Fractal wood burning is a form of pyrography art, creating Lichtenberg figures (intricate fern-like patterns) in wood using high voltage electricity. The electrical device used is commonly constructed using a high voltage transformer from a home microwave oven. This popular but dangerous art technique has resulted in electrical injuries with exceedingly high mortality rates. Presented is the case of a 37-year-old female artist who sustained an electrical injury from fractal wood burning resulting in full thickness burns to her hands. The burn wounds were reconstructed using a synthetic dermal substitute. This case report highlights the dangers associated with fractal burning, in particular the risk of high voltage electrical burns.

## Introduction

Fractal wood burning is a form of pyrography art, creating Lichtenberg figures (intricate fern-like patterns) in wood using high voltage electricity.^1^ It has recently gained popularity through social media platforms.^2^ The device used is commonly constructed using a high voltage transformer from a home microwave oven.^3^ This recently popular but incredibly dangerous art technique has resulted in electrical injuries with a mortality rate of at least 70%.^4^ This case describes a high voltage electrical burn sustained from fractal burning and two-staged reconstruction using a synthetic dermal substitute.

## Case

A 37-year-old, previously well female artist with a history of tobacco smoking and methamphetamine use, presented to a local district hospital following an electrical injury causing full thickness burns to her left thumb and right index finger. She had been creating pyrography art using a home-made wood fractal burning device to create Lichtenberg figures on wood panels. When switching on the device, the patient sustained a high voltage electrical injury. She reported tetany and loss of urinary continence, and a bystander removed the patient from contact with the device. The patient was transferred to a major tertiary emergency department and was initially managed as a low voltage electrical injury. Further history on arrival to the burns unit revealed the homemade device was created using a transformer from a household microwave oven, modifying it to produce a high voltage electrical current re-classifying the mechanism to a high voltage injury.

**Figure 1:**
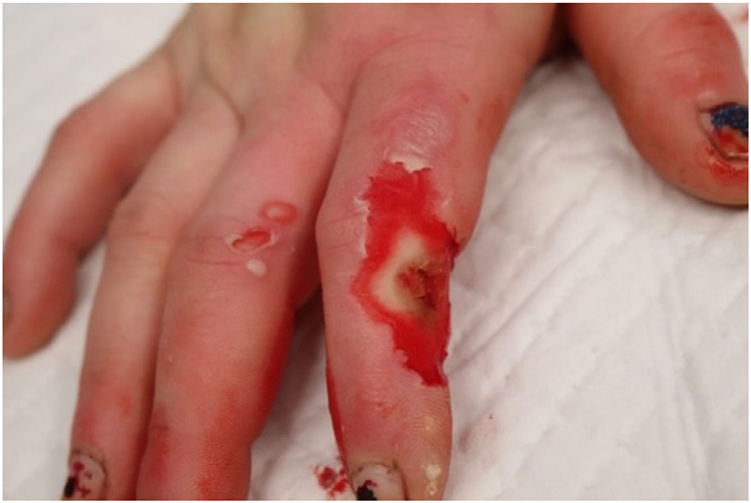
Initial debridement.

**Figure 2:**
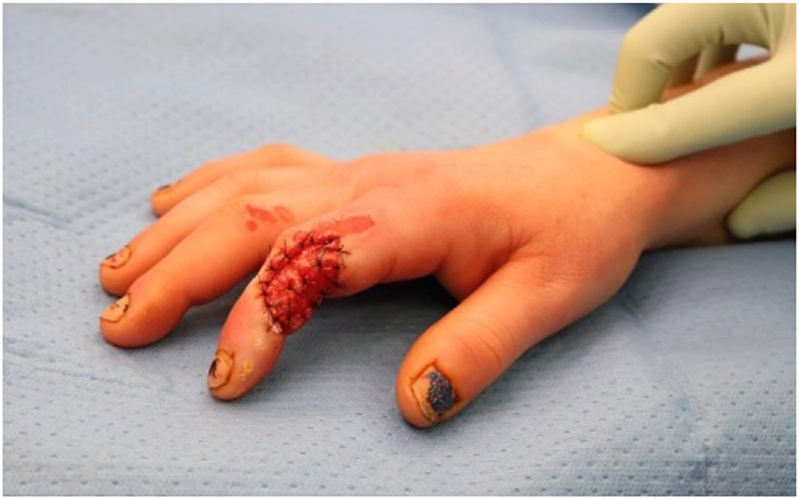
Dermal substitute application.

The patient was subsequently investigated with whole body computed tomography imaging for associated injuries and underwent cardiology consultation and trauma surveys with no abnormalities detected. On admission, creatinine kinase was 182 u/L (RR 0–150) and serial troponin levels were not elevated at 3 and 6 ng/L (RR < 12). Serial electrocardiography (ECG) demonstrated normal sinus rhythm. The patient was taken to the operating theatre one day following injury for tangential burn debridement. A full thickness burn was noted on the ulnar border of the left thumb measuring 2 × 1.5 cm and a 1 × 1 cm area of mid dermal burn on the right index finger were dressed with non-adhesive silver-based dressing. A 2 × 3 cm area of full thickness burn on the right index finger dorsoradial aspect middle phalanx was excised extensor tendon and bare lateral proximal interphalangeal joint capsule with identification and protection of the radial neurovascular bundle (Figure 1). A synthetic bilayered dermal substitute (Novosorb®BTM) was hand fenestrated and sutured to the wound (Figure 2) and dressed with a non-adhesive silver-based dressing.

The patient was discharged home one day post-operatively, with a plan for dermal substitute delamination and skin grafting 4 wk following application. However, the patient failed to attend multiple appointments therefore the second stage of surgery was delayed. Six weeks following initial debridement the synthetic dermal substitute was delaminated and a 0.012 split thickness skin graft from the right thigh was inset on top of a healthy dermal wound bed. Physiotherapists guided early hand mobilisation following inset of the synthetic dermal substitute and a good range of motion was maintained at the proximal interphalangeal joint throughout the dermal substitute integration period and graft healing.

**Figure 3:**
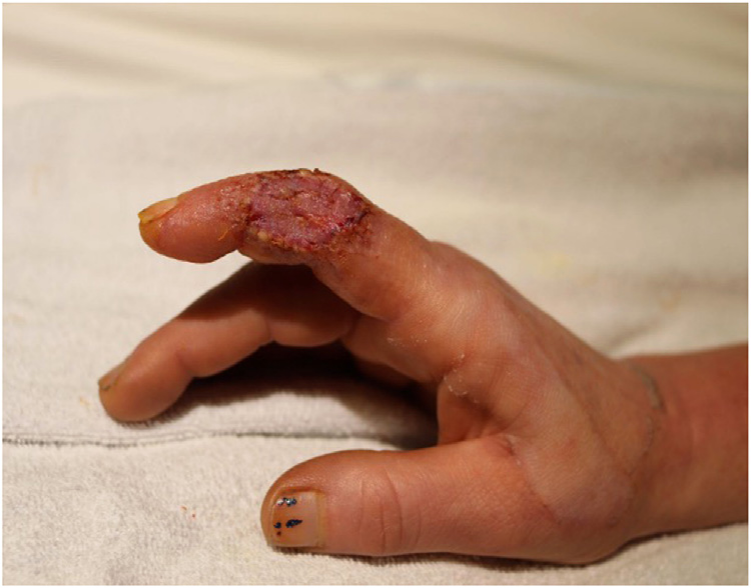
Following skin graft take.

The patient returned to the burns outpatient clinic for follow up and the site of electrical injury was observed to be healing well (Figure 3). She continued to create art with good dexterity, while avoiding the use of high voltage equipment.

## Discussion

Homemade equipment for fractal wood burning use transformers to amplify the voltage from a standard household current from 240 volts (V) to up to the extremes of 15,000 V.^4^ Some Australian state governments have prohibited the sale and use of fractal burning equipment, but in many areas of the country, its use is largely unregulated.^5^ High voltage electrical injuries may result in cardiac arrhythmias, head and spine trauma, organ failure and severe burns.^6^ Electrical burns are notoriously challenging to manage and can result in significant morbidity and mortality.^7^

When considering reconstructive options for treating full thickness electrical burns factors including patient compliance, lifestyle, site and extent of injury and comorbidities should be considered. Surgical options include skin grafting, provided a viable bed remains following debridement, local flaps of adjacent tissue to close the defect including advancement, rotation, or transposition flaps. In the case described, skin grafting was not an option given the presence of extensor tendon devoid of viable paratenon. Heterodigital flaps such as cross finger flaps could be considered but require a disabling period of immobilisation between stages.^8^ Dorsal metacarpal artery perforator-based flaps (DMCAP) are versatile options that could provide coverage to joints and have been successfully utilised for reconstruction of hand burn injuries.^9^ For larger defects, distant groin flaps or microsurgical free flap reconstruction may be indicated. The flaps described carry risk of failure, altered cosmesis, require longer operating times and patient compliance to ensure a good outcome, and carry increased risk for hand stiffness due to prolonged immobilisation to minimise flap vascular compromise in early healing.

Synthetic dermal substitutes offer an alternative solution to generate a neodermal scaffold bridging over avascular structures and allows for later simple skin grafting.^10^ Advantages of dermal substitutes include shorter operating times, ability for the patient to return home and commence daily activities or work and engage in hand therapy and early mobilisation to reduce stiffness. The patient presented demonstrated lack of follow up reliability making the dermal substitute a preferable option as the surrounding more superficial burn areas could heal conservatively allowing for a stable wound bed for subsequent graft take without compromise of hand function.

## Conclusion

This case report demonstrates a two-staged reconstruction of full-thickness electrical burns using a synthetic dermal substitute and split thickness skin graft. Public awareness of the potential dangers of creating fractal wood burning art should be raised to reduce the risk of future occurrences of these types of injuries.

## Patient consent

Informed written consent obtained from the patient for publication of case and clinical images.

## Funding

The authors received no financial support for the research, authorship and/or publication of this article.

## Ethical approval

Not required.

## Conflict of interest

The authors have no conflicts of interest to disclose.
